# Editorials

**Published:** 1890-01

**Authors:** 


### EDITORIALS.

THE EUROPEAN EPIDEMIC.—The epidemic of influenza now prevailing in Europe is being treated by our friends the allopaths in their usual scientific method. They spray the affected membrane with a solution of Quinine, freely and frequently applied, and give Quinine and other drugs internally. We can only sympathize with those who will have the effects of this treatment to overcome after the idiopathic affection has passed away. We are not in possession of sufficient data to enable us to say positively what will be the genius epidemicus, if we shall receive a visitation ; but, as Hahnemannians, we know that our patients will have no drug disease to battle with, and that when the original malady has gone they will be well. It now looks as though Camphor, Ars., Bry., and Nat-mur. will be called upon to assist in eradicating the trouble. G. H. C.

---

Scientific medicine, in the form of the Health Board of New York, recommends the following for the treatment of epidemic influenza: "Spray the affected membrane with a ten per cent. solution of Quinine freely and frequently, and take four or five times a day a pill made as follows: Quinine, 3 grs.; Camphor, ½ gr.; extract of Belladonna, ¼ gr." *The New York Medical Record* says: "Very many of the pharmacists about town have very properly refused to make up the prescription, and very many patients who believe in wholesale cheap prescribing may follow the directions to their sorrow." How fortunate for patients of regular medicine that there stand between the prescription and its compounding the pharmacist. A short time ago a druggist in this city showed us a prescription which if compounded according to the prescriber's directions the patient would have taken a poisonous dose with each spoonful. Compare this with the simple grandeur of Hahnemannian Homœopathy, and yet some prate of the schools coming closer together. To confirm one's belief in Homœopathy one need only familiarize himself with the best the modern allopath can do. For illustration place the following beside our therapeutics of typhoid fever, or any other disease, and then say which is the scientific: The professor of materia medica and therapeutics in the "College of Physicians and Surgeons," New York, in an article in the *Medical News*, Dec. 14th, 1889, entitled "A Contribution to the Therapeutics of Typhoid Fever," says—and he is here speaking for his own school—he knows nothing of Homœopathy, evidently:—"The results in the treatment of typhoid fever continue to be so bad in general in this country as to constitute a chronic opprobrium to the art of medicine here. * * * I believe, too, that the fact that we have not as yet arrived at anything at all resembling a general concensus of opinion [you need a law] regarding its treatment is in itself evidence that many cases must be improperly [*sic*] treated, and that in this way much of our high mortality is to be accounted for," and so on *ad nauseam*. Place by the side of this Dr. P. P. Wells' article on typhoid fever, published as a supplement to Vol. IV of this journal, and it will then be seen how closely we are coming together. On the one hand is mere empiricism, on the other there is what law makes necessary. We are not dependent upon the contingent.

---

"LA GRIPPE."—We trust our readers will report the results of their treatment in all cases of the prevailing epidemic which have come, and may yet come, under their care. We are able to record no ill effects after the first symptoms have passed away. On the other hand, our allopathic friends have reported many deaths from pneumonia and bronchial complications. To term these affections "complications" sounds nicely in the ears of the patients and their friends; but we should give them their proper place, and say they are due to the treatment. The *Medical Record* of New York, of January 4th, contains two articles on the subject. In these we find that "for headache, Antipyrin, ten to fifteen grains, with whisky, is administered. If the attack occurs in the morning a mild cathartic is administered, generally Calomel, in two to ten-grain doses. This is followed at night by a ten-grain Dover's powder and ten grains of Quinine. On the following day, when the catarrhal symptoms become marked, a pill of Morphine (gr. $\frac{1}{10}$), ext. Bellad. (gr. $\frac{1}{5}$), Camph. (gr. $\frac{1}{2}$), and Quin. (2 to 3 grs.), is given every three or four hours.

"When the bronchitis is the prominent feature, a mild expectorant mixture is given, containing syrs. Licorice, Squills, Senega, chloride of Ammonia, Morphine, etc."

This on January 4th, 1890. On December 21st, 1889, the same journal editorially says: "We notice with considerable surprise the publication in the daily papers of a prescription for the threatened epidemic of influenza, which is said to be by the sanction of the Board of Health. We trust we have been misinformed on the subject, *as the so-called remedy is not only of no good whatever* (we italicize), but its use as directed is *liable to be attended with great danger*. Here is the prescription in question: 'For Russian influenza or "grip"—spray the affected membrane with a ten per cent. solution of Quinine, freely and frequently, and take four or five times a day a pill made as follows: Quinine, 3 grains; Camphor, $\frac{1}{2}$ grain; extract of Belladonna, $\frac{1}{4}$ grain.'

"Very many of the pharmacists about town have very properly refused to make up this prescription, and very many patients who believe in wholesale cheap prescribing may follow the directions to their sorrow. We cannot believe that the publication of this prescription is made by the authority of the Health Board. If it is, the Board owes an explanation and apology to the profession and the public."

Is comment necessary? We have treated numerous cases of the affection, and have had no trouble whatever. We used the remedy indicated according to the homœopathic law, and in the potentized form only. We know that every Hahnemannian has done the same, and that patients have only praise for the good results. G. H. C.

---

Antipyrin, the present cureall of our friends the allopaths, is now being charged with sins even greater than have been attributed to Morphia, Chloral, Cocaine, and other infernal drugs. It is so soothing to the nerves that the habit once formed, as with the other drugs of that class, it becomes almost impossible to discontinue it. Notwithstanding this, there are few prescriptions now given for headache and neuralgic affections which do not contain antipyrin. It is an excellent substance for making work for the followers of Hahnemann. And now Antipyrin is recommended by the leading allopaths of Paris for use in the treatment of the prevailing epidemic influenza. They wish it known that the malady is not so benign as has been reported, as bronchitis and pneumonia may follow. Here is a query which any Hahnemannian can answer: How much of the condition following the primary attack is due to Antipyrin? We know that it is not necessary for us to dwell on this point. Any one who has practiced Homœopathy conscientiously will attest the fact that he rarely sees any of the so-called sequelæ of disease. Why? He treats his patients rationally. He gives them no crude drugs. He has no drug disease follow. Dear allopaths, study the *natural* history of pathological affections, and you will then soon have sufficient acuity of vision to see that your sequelæ of disease is drug disease, modified by the patient's constitutional condition. Make a note of this, you who take the name of "homœopathist" and imitate the druggers.

---

Intermittent Pulse.—Nature is coy and will not bear scrutiny. The more closely you scrutinize her workings of the animal economy the more she will punish you. These words occurred to us the other day when a patient came to us in much fear generated by being told by a strange physician that his pulse was intermittent, and that it indicated serious heart trouble.

As a consequence he had been closely watching his pulse, and was, and had been, in a constant state of fear.

After examining his heart, and assuring him that there was no disease there, and then giving a remedy to assist him in mastering the fear, we were gratified to find soon after that he had lost all sensations about the region of the heart.

Too often are patients thus made miserable by a superficial diagnosis. It should not be forgotten that intermittent pulse, without other signs of heart trouble, has little or no significance. Serious organic heart disease may exist and yet there will be no intermittency of the pulse. On the other hand, intermittent pulse may be constant and be of no serious import whatever. Intermittent pulse without organic heart changes is a mere nervous trick. In a large number of cases it arises from some depressing emotion, such as fear or grief or worry, and it is usually found to be a common symptom of shock to the nervous system. The honest physician, he who has the best interests of his patient at heart, will always "be sure he is right before he goes ahead." It is the part of wisdom never to give a prognosis until all the factors entering into the case are duly considered. Even then the power for good of Hahnemannian Homœopathy should be kept in view before any case is pronounced incurable.

G. H. C.

---

THE DEFINITION OF A HOMŒOPATHIC PHYSICIAN.—In the *New York Tribune* of December 19th is a report of the proceedings of The Homœopathic Medical Society of the County of New York. The subject under discussion related to the notorious differences concerning the Homœopathic Hospital board.

One of the most prominent of the members of the Society—a well-known homœopathic physician in New York City—made the following surprising statement:

"The definition of a homœopathic physician is: 'one who is a member of the Homœopathic County Medical Society.' No matter if I do have recourse to allopathic remedies, I am a homœopathic physician as long as I am a member of this Society. We should study pathology and surgery in common with allopaths, but the time is not come for us to be responsible for the actions of men not members of this Society."

According to this statement Homœopathy is not what is defined by Hahnemann; not what is defined by strictest logic, but what is indorsed by the New York Society!!

We always thought that Homœopathy consisted of the similar remedy, the single drug, and the minimum dose; but instead it is membership in the New York Society!

Why should any man wish to pass himself off as a homœopathic physician if he does not believe in the principles? Why should he not manfully renounce the title and fellowship with its followers and practice his own system after the example set by the *New York Medical Times?*

Statements like the one quoted made as they are by prominent men in practice, do much harm to those who are on the threshold of a homœopathic career, and discourage those who are struggling along toward the light.

The rank and file of the profession will not try to make homœopathic prescriptions. They will not believe it possible to control pain with the dynamized similar remedy in place of the ordinary and ruinous anodyne. Yet it can be done, *is* done constantly. But those who do it rarely take the time and pains to report their successes to the journals. So we earnestly appeal to our readers to supply us with all the clinical experience they can, that we may be aided in our efforts to teach Hahnemannian Homœopathy, and give aid and comfort to all who are willing to stand by the cause, all who are disheartened by the follies of those who assume to be leaders in the profession.

W. M. J.

---

Nothing New.—The idea of germs acting as causes of so many diseases is an idea which has become almost universal in these days and is generally considered to be of modern origin. Yet we find it advanced in the year 1666 by a Dr. Charlton, who believed germs to be the cause of the great plague which visited England in 1665. These germs, it was said, laid microscopic eggs, which being snuffed up by a dog, caused all the symptoms of the plague.

---

Eclecticism.—There is a so-called homœopathic physician of some fame (among those who do not know what Homœopathy is), who has been heard to say: "I practice Homœopathy, but when I have a serious case, or have failed in my prescription, I resort to allopathy." If this be "Homœopathy," we would rather *try* allopathy in the beginning. But there is no need to resort to allopathy while we have the "light" descended from our grand leader Hahnemann, which will never fail us, unless darkened by ignorance and cowardice.

To Our Correspondents.—We beg the indulgence of all who write letters to us for the seeming neglect to answer them. We are overwhelmed with work. It is impossible for us to keep up with all the demands upon our time, and so our correspondence falls into arrears. We desire, however, to assure our readers that no communication sent us will be totally ignored. Though an answer may be long delayed, it will be sent ultimately. We therefore hope that our subscribers will continue to favor us with their epistles.

---

Disinfectants.—When will people in general, and physicians in particular, give up the fallacious, so-called disinfectants? Even at the present day one's olfactory bulb is made to revolt by the odors of Carbolic acid. It is our duty always to warn patients against the use of these nasty substances. They do no good, can only do harm, and cost a deal more than the only true disinfectant, fresh air.

